# LncRNA-TUG1 promotes the progression of infantile hemangioma by regulating miR-137/IGFBP5 axis

**DOI:** 10.1186/s40246-021-00349-w

**Published:** 2021-08-06

**Authors:** Lili Zhou, Xiao Jia, Xiangzheng Yang

**Affiliations:** 1 Department of Pediatrics, Beijing University of Chinese Medicine Shenzhen Hospital (Longgang), No. 1 Dayun Road, Shenzhen City, Guangdong Province 518000 People’s Republic of China; 2 Department of Orthopedics, Gansu Provincial Hospital of TCM, Lanzhou City, Gansu Province 730050 People’s Republic of China

**Keywords:** Infantile hemangioma, TUG1, miR137, IGFBP5, Tumorigenesis

## Abstract

**Background:**

Previous studies indicated that lncRNA taurine upregulated gene 1 (TUG1) played essential roles in human cancers. This study aimed to investigate its function in infantile hemangioma (IH).

**Methods:**

A total of 30 pairs of clinical infantile specimens were used in this study. The expression of TUG1 in IH tissues was assessed by quantitative reverse transcriptase PCR (qRT-PCR). Two short hairpin RNA targeting TUG1 (sh-TUG1-1 and sh-TUG1-2) were transfected into hemangioma-derived endothelial cells, HemECs, to block its expression. The effects of TUG1 on HemECs were evaluated by Cell Counting Kit-8 (CCK-8), colony formation assay, wound healing assay, and Transwell assay. The underlying molecular mechanism of TUG1 was investigated by Starbase prediction and luciferase reporter assay and further determined by loss- and gain-of-function approaches. In addition, the role of TUG1 on tumorigenesis of HemECs was confirmed in an in vivo mouse model.

**Results:**

TUG1 was significantly upregulated in infant hemangioma tissues compared with normal adjacent subcutaneous tissues. The loss- and gain-of-function approaches indicated that TUG1 overexpression promoted proliferation, migration, and invasion of HemECs in vitro, and TUG1 knockdown inhibited the tumorigenesis of HemECs in vivo. Specifically, TUG1 could compete with IGFBP5 for miR137 binding. Rescue experiments further confirmed the role of the TUG1/miR137/IGFBP5 axis in HemECs.

**Conclusion:**

TUG1 was closely associated with the progression of IH by regulating the miR-137/IGFBP5 axis, which might be a potential target for IH treatment.

## Background

Infantile hemangioma (IH) is one of the common malignancies, leading to higher mortality in the infant population [1]. Previous reports suggest that IH occurs in up to 10% of non-Hispanic Caucasian infants and is more prevalent in premature, low birth weight, and multiple-birth babies [2, 3]. Although several key genes have been revealed to be involved in IH development [4, 5], the mechanisms underlying IH progression are still not completely known.

Long noncoding RNAs (lncRNAs) are a widely known class of noncoding RNAs with more than 200 nucleotides in length and often regulate the expression of downstream genes at the post-transcriptional level [6]. Increasing evidence indicates that lncRNAs possess various regulatory functions in cancer progression, including proliferation, migration, and invasion [7]. lncRNA taurine upregulated gene 1 (TUG1) is one of the first identified lncRNAs associated with human diseases [8]. For example, TUG1 promotes cell proliferation and inhibits apoptosis by targeting AURKA in epithelial ovarian cancer [9]. TUG1 promotes proliferation, migration, and epithelial-mesenchymal transition of papillary thyroid cancer cells by targeting miR-145 [10]. TUG1 promotes the progression of osteosarcoma through modulating RUNX2 expression [11]. In addition, TUG1 is also associated with the development of breast cancer [12], colorectal cancer [13], and gastric cancer [14]. These reports confirmed the crucial roles of TUG1 in human cancers. However, the function of TUG1 in IH remains unclear.

MicroRNAs (miRNAs) are also noncoding RNAs with approximately 22 nucleotides in length and can bind to the 3′ UTR of their target genes to inhibit their translation [15]. Many studies have demonstrated that miRNAs affect the progression of IH and may be considered as potential biomarkers. Downregulation of miR-382 inhibits IH progression by regulating the PTEN-mediated AKT/mTOR signaling pathway [16]. MiR-143 acts as a suppressor of IH by targeting Bcl-2 [16]. MiR-137 is a rarely reported microRNA that has been only studied in melanoma. Qi et al. found that miR-137 is downregulated in melanoma cell lines and tissues, and knockdown of miR-137 inhibits melanoma cell invasion and migration [17]. In addition, one recent study demonstrated that miR-137 was a target of TUG1, and this axis closely participated in the migration and invasion of hepatocellular carcinoma cells [18].

Insulin-like growth factor-binding protein 5 (IGFBP5), a member of the insulin superfamily of growth-promoting peptides, has been revealed to be the most abundant polypeptide growth factor [19]. Its role in tumors has been extensively studied. Some showed that IGFBP5 played a negative role in tumor progression by suppressing tumor cell proliferation and metastasis [20, 21]. Rho et al. showed that IGFBP5 expression prevented tumor growth and inhibited tumor vascularity in a xenograft model of human ovarian cancer [20]. Butt et al. showed that IGFBP5 promoted breast cancer cell apoptosis via Bcl-2 in the intrinsic apoptotic pathway [22]. However, other studies showed that IGFBP5 played a positive role in tumor progression by promoting tumor cell survival and migration [23–25]. In addition, IGFBP5 acts as an oncogene in different cancers such as gastric cancer [26], colorectal cancer [27], prostate cancer [28], glioblastoma [29], human melanoma [30], and breast cancer [23]. To date, it is not clear that the role and exact mechanisms of IGFBP5 in IH progression. Previous studies reported that miR-137 suppressed cell proliferation and migration of vascular smooth muscle cells by targeting IGFBP-5 [31], suggesting that IGFBP5 was a target of miR-137.

In the present study, we found that TUG1 was significantly upregulated in infant hemangioma tissues, especially in proliferating-phase hemangioma tissues. Knockdown of TUG1 efficiently inhibited proliferation, migration, and invasion of HemECs in vitro and obviously reduced tumorigenesis of HemECs in vivo. Moreover, TUG1 was found to regulate IGFBP5 expression by directly sponging miR-137, and the miR-137/IGFBP5 axis mediated the roles of TUG1 in IH progression.

## Methods

### Clinical specimens

A total of 30 pairs of clinical infantile specimens, including hemangioma tissues and normal adjacent subcutaneous tissues surrounding hemangiomas, were collected from 30 infantile hemangioma patients at Beijing University of Chinese Medicine Shenzhen Hospital (Longgang) from 2015 to 2019. The specimens were immediately frozen at − 80 °C or in liquid nitrogen until further analysis. Among them, 15 were from patients with proliferative hemangiomas (13 females and 2 males; average age 6 months) and 15 from patients with involuting hemangiomas (10 females and 5 males; average age 7 months). The parents/legal guardians of all participants have signed the informed consent. This study was approved by the Human Ethics Committee of Beijing University of Chinese Medicine Shenzhen Hospital (Longgang) and conducted in accordance with the guidelines of the Declaration of Helsinki.

### Cell isolation and culture

Hemangioma-derived endothelial cells, HemECs, were isolated from infant hemangioma tissues at the proliferating phase as previously described [32]. HEK-293T cells (Life Technologies) were used for luciferase reporter assay. HemECs and HEK-293 T cells were cultured in DMEM supplemented with 10% fetal bovine serum (FBS) with 5% CO_2_ at 37 °C.

### Cell transfection

Two short hairpin RNA targeting TUG1, sh-TUG1-1 (5′-GACTACCTTCCCTGTGCTATT-3′) and sh-TUG1-2 (5′-TGCAACTGACATGCTAG CCGA-3′) and the non-targeting sh-RNA (sh-NC) were obtained from GenePharma (Shanghai, China). To overexpress TUG1 and IGFBP5, the full lengths of TUG1 and IGFBP5 were synthesized from GenePharma (Shanghai, China) and cloned into the pcDNA3.1 vector (GenePharma, Shanghai, China) to generate TUG1 and IGFBP5 overexpressing vector, respectively. The empty vector pcDNA3.1 was used as the negative control. MiR-137 mimics, NC mimics, miR-137 inhibitor, and NC inhibitor were purchased from GenePharma (Shanghai, China), and their sequences are shown in Table 1. Cell transfection was performed using Lipofectamine 3000 (Thermo Fisher Scientific, Inc.) according to the manufacturer’s protocol.

**Table 1:** The sequences of mimics and inhibitor

	Sense	Antisense
MiR-137 mimics	5′-UUAUUGCUUAAGAAUACGCGUAG-3′	5′-ACGCGUAUUCUUAAGCAAUAATT-3′
NC mimics	5′-UUGUCCGAACGUGUCACGUTT-3′	5′-ACGUGACACGUUCGGAGAATT-3′
miR-137 inhibitor	5′-CUACGCGUAUUCUUAAGCAAUAA-3′	
NC inhibitor	5′-CAGUACUUUUGUGUAGUACAA-3′	

### Cell proliferation

Approximately 1 × 10^4^ HemECs with 48 h of transfection were seeded each well of 96-well plates. At 24, 48, 72, and 96 h after inoculation, 10 μl of Cell Counting Kit-8 (CCK-8) solution (Dojindo Molecular Technologies, Inc.) was added into each well, and the plates were incubated for 2 h. After that, cell viability was determined by measuring the absorbance at 450 nm using a microplate reader (Bio-Rad, Hercules, CA, USA).

### Colony formation assay

Cell proliferation was also evaluated by colony formation assay. In brief, approximately 1000 HemECs were seeded into 6-well plates and cultured for 14 days. Cells were fixed in formalin and stained with 0.1% crystal violet (Solarbio), and then the colonies were photographed and counted under a digital camera.

### Migration assay

Approximately 1 × 10^4^ transfected or un-transfected HemECs/well were seeded into 6-well plates and cultured for 24 h. The cell monolayers were scratched using a 100-μl micropipette tip. After the floating cells and debris cells were washed out with PBS, cell migration was observed and photographed under a light microscope (Olympus, Tokyo, Japan) at 0 h and 48 h.

### Invasion assay

Cell invasion was evaluated by using the 8-mm pore-size Transwell filters (Costar, Corning Incorporated) as previously described [33]. Briefly, 1 × 10^4^ HemECs/well in 100 μL of FBS-free DMEM were placed on the upper chambers of the Transwell in a 24-well plate, and the lower chambers were filled with 300 μL of DMEM with 10% FBS. After 24 h of incubation, the cells invaded the lower surface of the filter were stained using crystal violet staining, counted, and photographed using a light microscope (Olympus, Tokyo, Japan).

### Quantitative real-time PCR (qRT-PCR)

Total RNA of tissues and cultured cells was extracted using Trizol (Thermo Fisher Scientific). About 1 μg of RNA was reverse transcribed into cDNA using the Reverse Transcription Kit (Takara, Dalian, China). The fold expression change of target genes was analyzed on an ABI 7500 PCR system (Applied Biosystems) using the Power SYBR™ Green PCR master mix (Thermo Fisher Scientific, Inc.). U6 and GAPDH were used as the internal control for miR-137 and TUG1/IGFBP5, respectively. Data were calculated using the 2^−ΔΔCt^ method. The primer sequences are shown in Table 2.

**Table 2:** The sequences of primers

	Forward	Reverse
TUG1	5′-CTGAAGAAAGGCAACATC-3′	5′-GTAGGCTACTACAGGATTTG-3′
miR-137	5′-CGGGCTTATTGCTTAAGAATA-3′	5′-GCAGGGTCCGAGGTATTC-3′
U6	5′-CTCGCTTCGGCAGCACA-3′	5′-AACGCTTCACGAATTTGCGT-3′
IGFBP5	5′-GCACCTGAGATGAGACAGGA-3′	5′-TGTAGAATCCTTTGCGGTCA-3′
GAPDH	5′-CGGACCAATACGACCAAATCCG-3′	5′-AGCCACATCGCTCAGACACC-3′

### Western blot

Total protein samples of tissues and cultured cells were extracted using RIPA lysis buffer (Beyotime Institute of Biotechnology). Approximately 10 μg protein samples were separated on 12% SDS-PAGE and transferred onto PVDF membranes. After blocked with 5% skimmed milk, the membranes were incubated with primary antibodies against IGFBP5 (Abcam, ab254324. 1:500), Ki67 (Abcam, ab15580. 1:500), and GAPDH (Abcam, ab9485, 1:1000) overnight at 4 °C. On the following day, the membranes were exposed to horseradish peroxidase-conjugated secondary antibody for 2 h. The signals were detected using an enhanced chemiluminescence detection kit (ECL, Bio-Rad, Hercules, CA, USA).

### Luciferase reporter assay

The putative binding sites between TUG1 and miR-137 and between miR-137 and IGFBP5 were predicted by Starbase. The fragments of TUG1 and IGFBP5 containing the wild type (WT) or mutant type (MUT) miR-137 binding site were obtained from GenePharma (Shanghai, China) and inserted into pmirGLO luciferase vector (GeneCreat, China) to generate the recombinant vector TUG1/IGFBP5 3′-UTR WT/MUT. HEK-293T cells were co-transfected with the recombinant vectors and miR-137 mimics or NC mimics using Lipofectamine 3000. At 48 h of transfection, the relative luciferase reporter activity was detected using the dual luciferase reporter system (Promega).

### In situ hybridization (ISH)

The hemangioma tissue and adjacent normal tissues were fixed in 4% formalin, embedded in paraffin, and cut into 5-μm sections. The Digoxin-labeled ISH probe for TUG1 was synthesized by GenePharma (Shanghai, China). The ISH assay was performed using the Enhanced Sensitive ISH Detection Kit (POD) (Boster Biotechnology) according to the manufacturer’s protocol. Subsequently, the sections were counterstained by hematoxylin for 90 s. After drying, the sections were photographed using a Zeiss invert microscope (CarlZeiss, Hallbergnoos, Germany).

### Immunofluorescence

IGFBP5 expression in HemECs was evaluated by immunofluorescence using an anti-IGFBP5 antibody. In brief, transfected or un-transfected HemECs were permeabilized in 0.1% Triton X-100 and incubated with anti-IGFBP5 primary antibody (Abcam, ab254324. 1:500) overnight at 4 °C. Cells were then incubated with FITC-conjugated goat anti-rabbit secondary antibody (Abcam, ab6717, 1:1000) at room temperature for 2 h. Nuclei were stained with DAPI (Boster Biotechnology). The stained cells were imaged using a Zeiss invert microscope (CarlZeiss, Hallbergnoos, Germany).

### In vivo model

BALB/c nude mice (male, 5-week-old) were purchased from Beijing Vital River Laboratory Animal Technology Co., Ltd. Then, 100 μl PBS containing 5 × 10^6^ sh-TUG1-1 or sh-NC transfected HemECs cells were subcutaneously inoculated into BALB/c nude mice as previously described [34] with five mice in each group. Tumor volume was evaluated every week for a total of 5 weeks based on the formula: length × width^2^/2. On day 35, mice were sacrificed by cervical dislocation, and the tumors were collected and weighted. All animal experiments were approved by the Animal Ethics Committee of Beijing University of Chinese Medicine Shenzhen Hospital (Longgang).

### Immunohistochemistry (IHC) assay

The tumors from the in vivo model were fixed in 4% formalin, embedded in paraffin, and cut into 3-μm sections. The sections were incubated with anti-Ki67 antibody (Abcam, ab15580. 1:500) at 4 °C overnight, followed by 2 h incubation with the secondary antibody working solution (Dako, GK500705). Subsequently, Ki-67 expression in tumor tissues was observed using a DAB Substrate Kit (Invitrogen, USA) according to the manufacturer’s protocol.

### Statistical analysis

Data were presented as mean ± standard deviation (SD) and statistically analyzed using SPSS 19.0 (IMB Corp.). Spearman’s correlation analysis was used to evaluate the relationship between the level of TUG1 and miR-137, as well as miR-137 and IGFBP5 in infant hemangioma tissues. Differences between two groups were tested by Student’s *t* test, and differences among multiple groups were determined by one-way ANOVA followed by Tukey’s multiple comparisons test. A *p* < 0.05 was considered a significant threshold, and a *p* < 0.01 was considered an extremely significant threshold.

## Results

### TUG1 was highly expressed in IH tissues

**Fig. 1:**
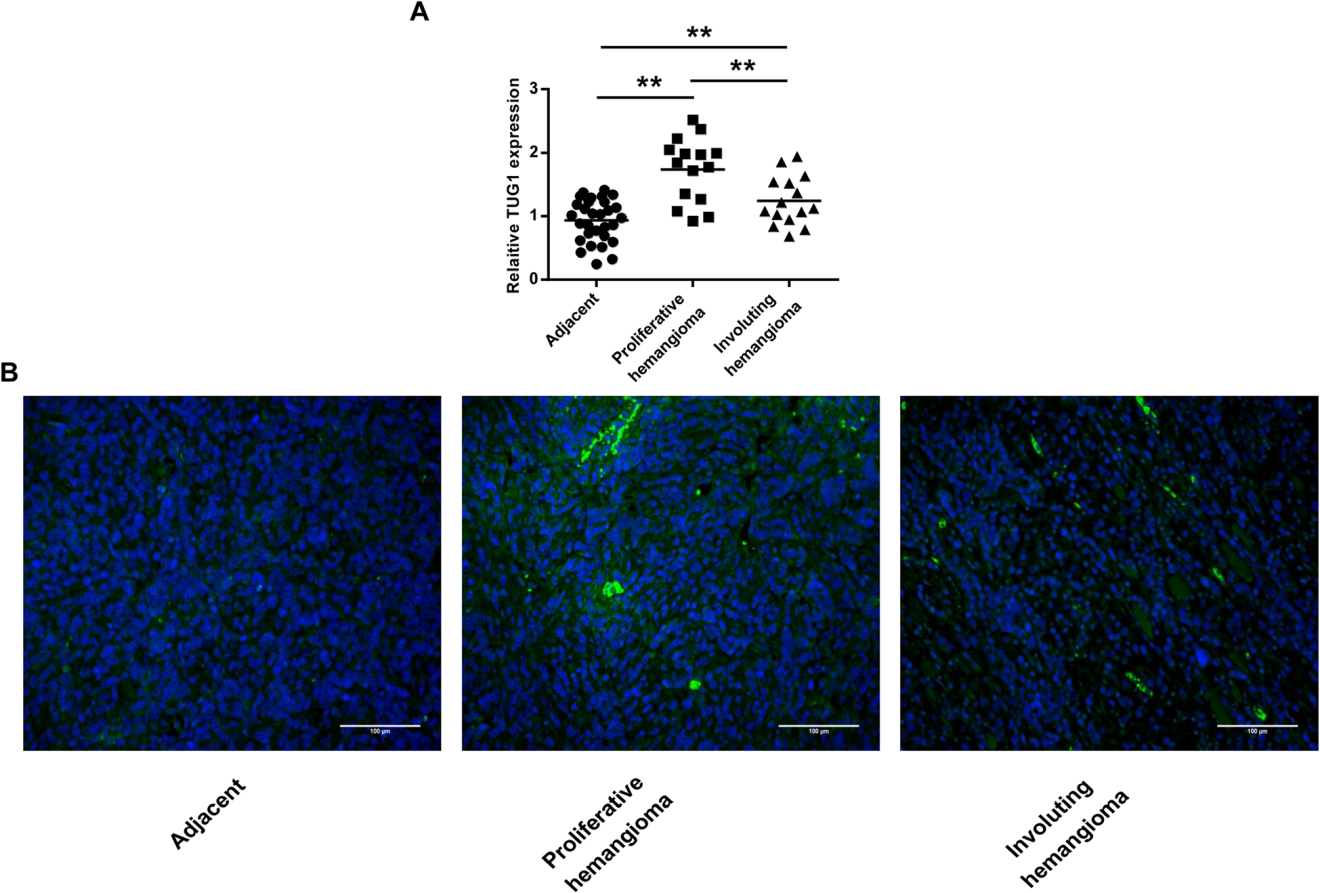
TUG1 was highly expressed in IH tissues. **A** TUG1 expression in proliferative-phase hemangioma tissues (*n* = 15), involuting-phase hemangioma tissues (*n* = 15), and adjacent normal subcutaneous tissues (*n* = 30) was assessed by qRT-PCR. **B** TUG1 expression in infant hemangioma tissues was evaluated by ISH assay. Magnification, × 200; scale bar = 100 μm. Data were expressed as mean ± SD. ** *P* < 0.01

To explore the role of TUG1 in IH progression, we firstly detected TUG1 expression in IH tissues and found that TUG1 was significantly upregulated in IH tissues compared with the adjacent normal subcutaneous tissues (*p* < 0.01 for proliferating-phase hemangioma tissues and *p* < 0.01 for involuting-phase hemangioma tissues). In addition, TUG1 expression was significantly higher in the proliferating-phase hemangioma tissues than in the involuting-phase hemangioma tissues (*p* < 0.01) (Fig. 1A). Meanwhile, TUG1 expression in IH tissues was also evaluated by ISH assay. The results showed that TUG1 was upregulated in IH tissues, especially in the proliferative-phase hemangioma tissues (Fig. 1B). These results indicate that TUG1 may play a potential oncogenic role in IH progression.

### TUG1 knockdown inhibited HemEC proliferation, migration, and invasion in vitro

**Fig. 2:**
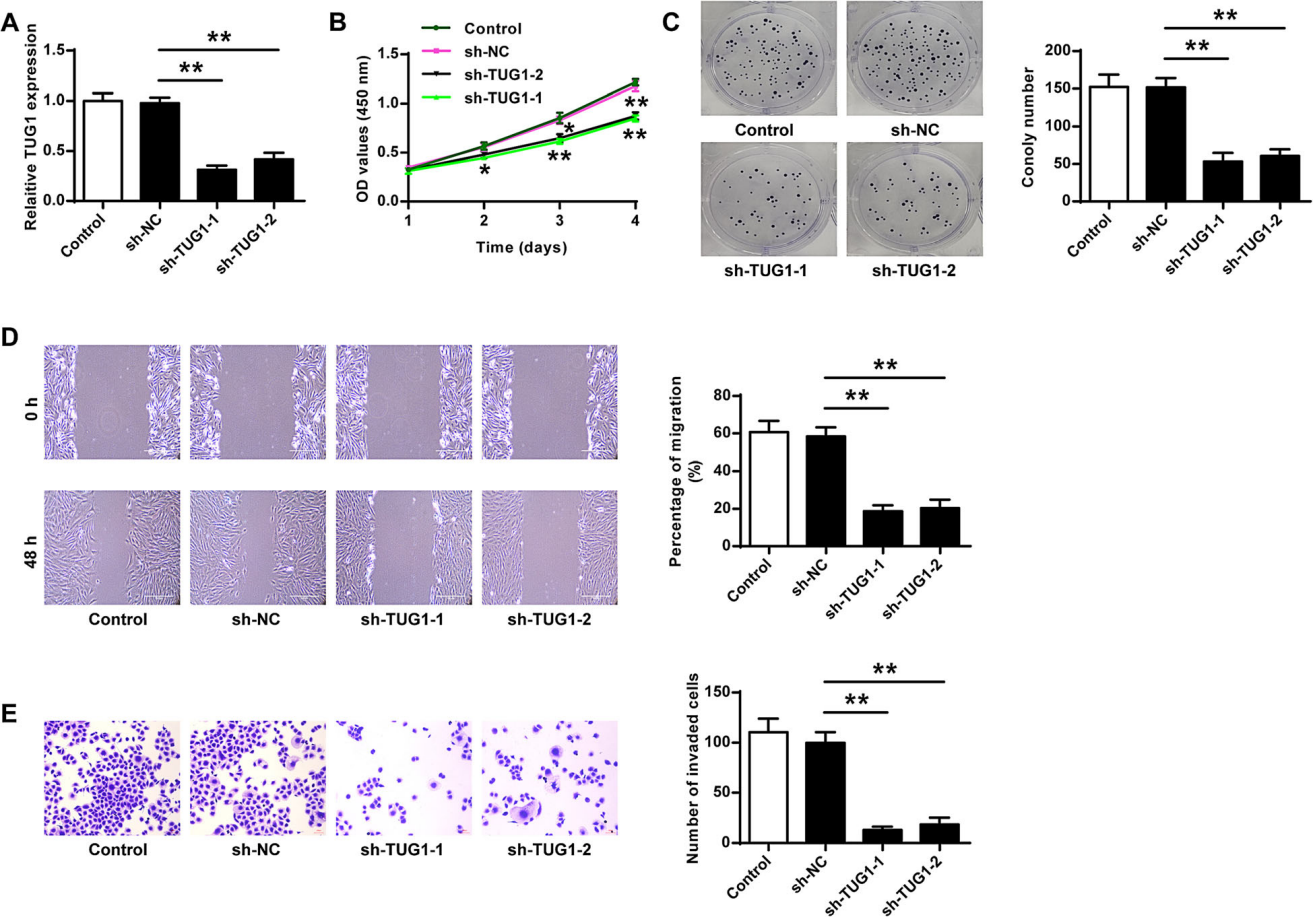
TUG1 knockdown inhibited proliferation, migration, and invasion of HemECs in vitro. HemECs were transfected with sh-TUG1-1, sh-TUG1-2, or sh-NC. **A** The transfection efficiency was confirmed by qRT-PCR. **B**, **C** Cell proliferation was evaluated by CCK-8 assay (**B**) and colony formation assay (**C**). **D** Cell migration was evaluated by the wound healing assay. **E** Cell invasion was evaluated by Transwell assay. Data were derived from three independent experiments and expressed as mean ± SD. * *P* < 0.05, ** *P* < 0.01

To determine the oncogenic role of TUG1 in IH, two sh-RNAs targeting TUG1 were transfected into HemECs. qRT-PCR confirmed that sh-TUG1-1 exhibited the best silencing effect (*p* < 0.01, Fig. 2A). As shown in Fig. 2B and C, both sh-TUG1-1 and sh-TUG1-2 efficiently reduced the proliferation of HemECs compared with the negative control sh-NC (sh-TUG1-1: *p* < 0.05 at 48 h, *p* < 0.01 at 72 h, *p* < 0.01 at 96 h; sh-TUG1-2: *p* < 0.05 at 48 h, *p* < 0.05 at 72 h, *p* < 0.01 at 96 h, Fig. 2B; both *p* < 0.01, Fig. 2C). The wound healing assay results showed that both sh-TUG1-1 and sh-TUG1-2 significantly inhibited the migration ability of HemECs compared with sh-NC (both p < 0.01, Fig. 2D). In addition, both sh-TUG1-1 and sh-TUG1-2 significantly inhibited the invasion capacity of HemECs compared with sh-NC (both *p* < 0.01, Fig. 2E). These observations suggested that TUG1 knockdown inhibited the proliferation, migration, and invasion of HemECs in vitro.

### TUG1 acted as a sponge of miR-137

**Fig. 3:**
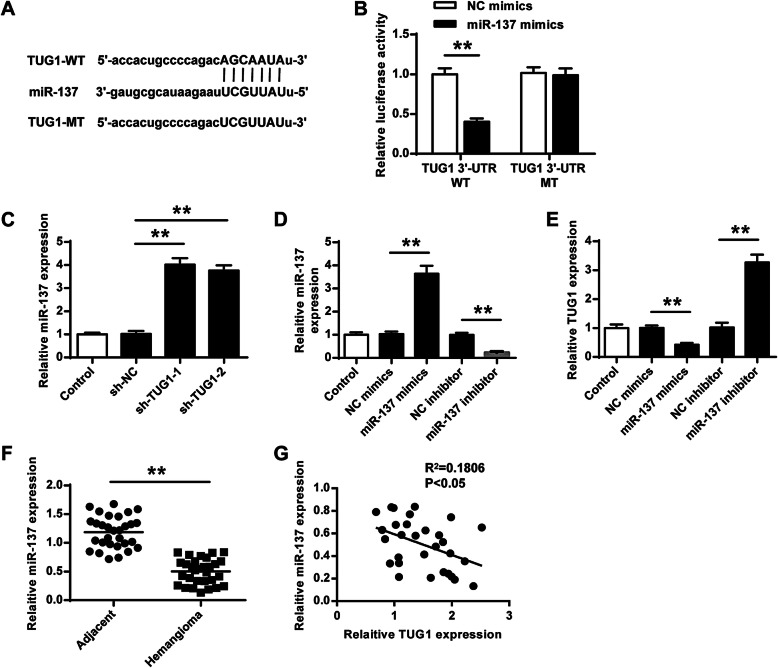
TUG1 acted as a sponge of miR-137. **A** The putative binding site between TUG1 and miR137 was predicted by Starbase. **B** The relative luciferase activity of TUG1 3′-UTR WT/MUT in HEK-293T cells was detected by the dual luciferase reporter assay. **C** HemECs were transfected with sh-TUG1-1, sh-TUG1-2, or sh-NC. MiR-137 expression was assessed by qRT-PCR. **D**, **E** HemECs were transfected with miR-137 mimics/inhibitor, and corresponding negative controls. The expression of miR-137 (**D**) and TUG1 (**E**) was evaluated by qRT-PCR. **F** MiR-137 expression in infant hemangioma tissues (*n* = 30) and adjacent normal subcutaneous tissues (*n* = 30) was assessed by qRT-PCR. **G** The correlation between the level of TUG1 and miR-137 in infant hemangioma tissues (*n* = 30) was evaluated by Spearman’s correlation analysis. Data were derived from three independent experiments and expressed as mean ± SD. * *P* < 0.01

To explore the mechanism of TUG1, Starbase (http://starbase.sysu.edu.cn/index.php) was used, and the prediction suggested that miR-137 might bind to the 3′-UTR of TUG1 (Fig. 3A). A luciferase reporter assay was performed to determine their interaction, and the results indicated that miR-137 overexpression significantly reduced the relative luciferase activity of TUG1 3′-UTR WT compared with NC mimics (*p* < 0.01) but did not alter the luciferase activity of TUG1 3′-UTR MUT (Fig. 3B). Meanwhile, TUG1 knockdown in HemECs by transfection of sh-TUG1-1/2 significantly increased miR-137 expression compared with sh-NC (both *p* < 0.01, Fig. 3C). Next, miR-137 mimics/inhibitor and corresponding negative controls were transfected into HemECs, and the transfection efficiencies were confirmed by qRT-PCR (*p* < 0.01, miR-137 mimics vs. NC mimics; *p* < 0.01, miR137 inhibitor vs. NC inhibitor; Fig. 3D). The results of qRT-PCR showed that the transfection of miR-137 mimics significantly reduced TUG1 expression compared with NC mimics (*p* < 0.01), and the transfection of miR-137-inhibitor significantly increased TUG1 expression compared with NC inhibitor (*p* < 0.01, Fig. 3E). Moreover, miR-137 expression was markedly downregulated in infant hemangioma tissues than in adjacent normal subcutaneous tissues (*p* < 0.01, Fig. 3F). Spearman’s correlation analysis indicated that TUG1 levels were negatively correlated to miR-137 levels in infant hemangioma tissues (*p* < 0.05, *R*^2^ = 0.1806, Fig. 3G). These results indicated that TUG1 served as a sponge of miR-137 in IH.

### MiR-137 inhibited HemEC proliferation, migration, and invasion by regulating IGFBP5

**Fig. 4:**
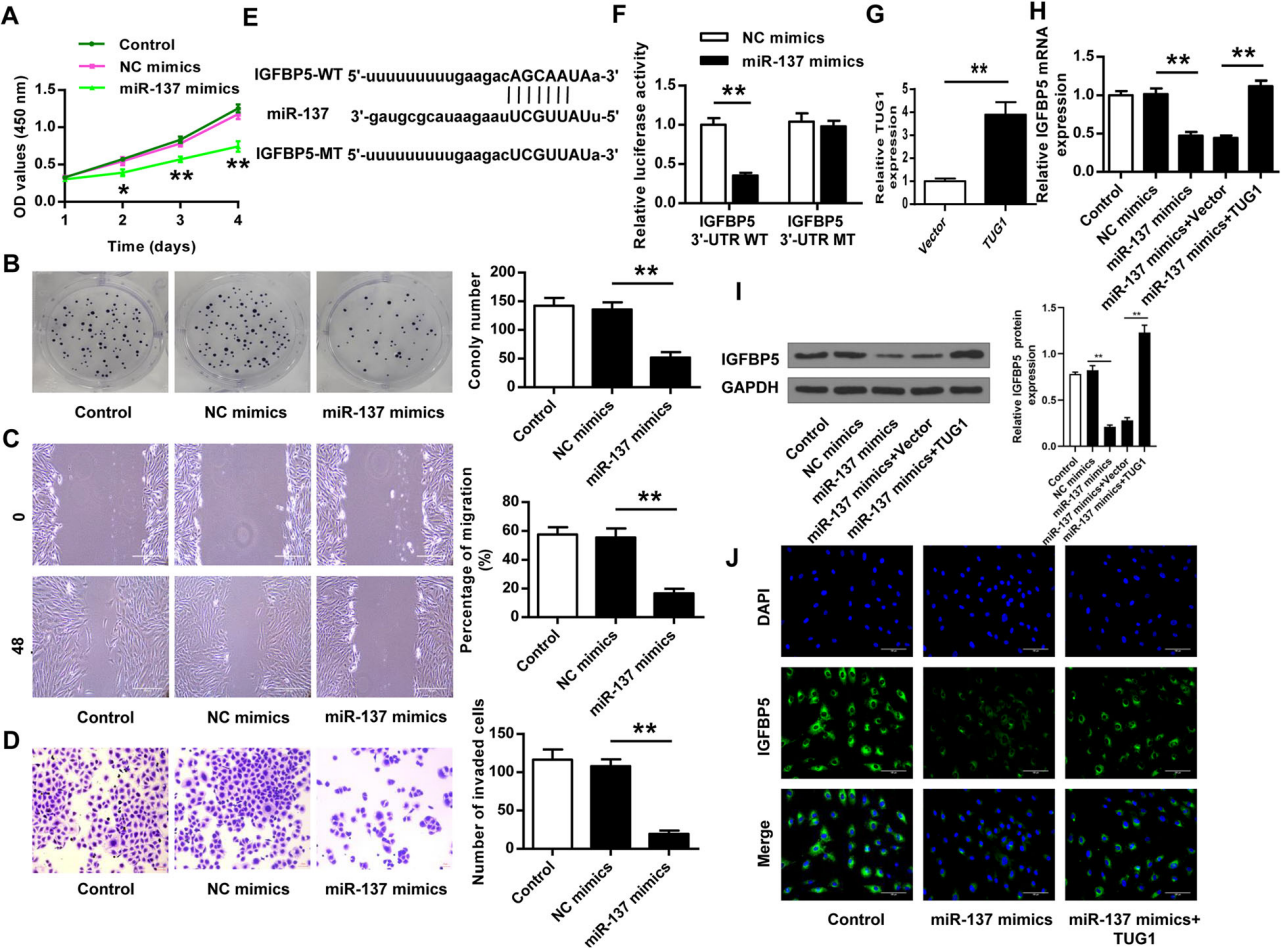
MiR-137 inhibited HemEC proliferation, migration, and invasion via regulating IGFBP5. **A**–**D** HemECs were transfected with miR-137 mimics and NC mimics. Cell proliferation was evaluated by CCK-8 assay (**A**) and colony formation assay (**B**). **C** Cell migration was evaluated by the wound healing assay. **D** Cell invasion was evaluated by Transwell assay. **E** The interactional region between miR-137 and IGFBP5 was predicted by Starbase. **F** The relative luciferase activity of IGFBP5 3′-UTR WT/MUT in HEK-293T cells was detected by the dual luciferase reporter assay. **G** HemECs were transfected with TUG1 (the overexpressing vector for TUG1) or empty vector pcDNA3.1. TUG1 expression was evaluated by qRT-PCR. **H**–**J** HemECs were transfected with miR-137 mimics, NC mimics, or co-transfected with miR-137 mimics and the empty vector, or miR137 mimics and TUG1 (the overexpressing vector for TUG1). IGFBP5 expression was evaluated by qRT-PCR (**H**) and Western blot (**I**). **J** IGFBP5 expression was evaluated by immunofluorescence using an anti-IGFBP5 antibody. Magnification, × 200; scale bar = 100 μm. Data were derived from three independent experiments and expressed as mean ± SD. * *P* < 0.05, ** *P* < 0.01

To further investigate the mechanism underlying the roles of TUG1 in IH, HemECs were transfected with miR-137 mimics and NC mimics, and HemEC proliferation was evaluated by CCK-8 assay (Fig. 4A) and colony formation assay (Fig. 4B). The results showed that miR137 overexpression significantly inhibited HemEC proliferation compared with NC mimics (*p* < 0.05 at 48 h, *p* < 0.01 at 72 h, *p* < 0.01 at 96 h, Fig. 4A; *p* < 0.01, Fig. 4B). Moreover, wound healing assay and Transwell assay showed that miR-137 overexpression significantly inhibited the migration and invasion of HemECs compared with NC mimics (both *p* < 0.01, Fig. 4C and D). The prediction using Starbase showed that there was a putative binding site between miR-137 and IGFBP5 (Fig. 4E), suggesting that IGFBP5 might be a target of miR-137. Luciferase reporter assay in HEK-293T cells indicated that transfection of miR-137 mimics significantly reduced the relative activity of IGFBP5 3′-UTR WT (*p* < 0.01), but not IGFBP5 3′-UTR MUT, compared with NC mimics (Fig. 4F). HemECs were transfected with the overexpressing vector for TUG1 (TUG1), and the transfection efficiency was confirmed by qRT-PCR (*p* < 0.01, Fig. 4G). The recuse experiment results showed that overexpression of miR-137 mimics reduced IGFBP5 expression compared with NC mimics (*p* < 0.01), and co-transfection of miR-137 mimics and TUG1 obviously reversed the inhibitory effect of miR-137 mimics (*p* < 0.01, Fig. 4H and I). In addition, IGFBP5 expression in HemECs was also evaluated by immunofluorescence using an anti-IGFBP5 antibody, and the results showed that the transfection of miR-137 mimics obviously reduced IGFBP5 expression, and co-transfection of miR-137 mimics and TUG1 reversed the inhibitory effect of miR-137 mimics (Fig. 4J). These results suggested that miR-137 inhibited the proliferation, migration, and invasion of HemECs via regulating IGFBP5.

### MiR-137 mediated the role of TUG1 in HemECs

**Fig. 5:**
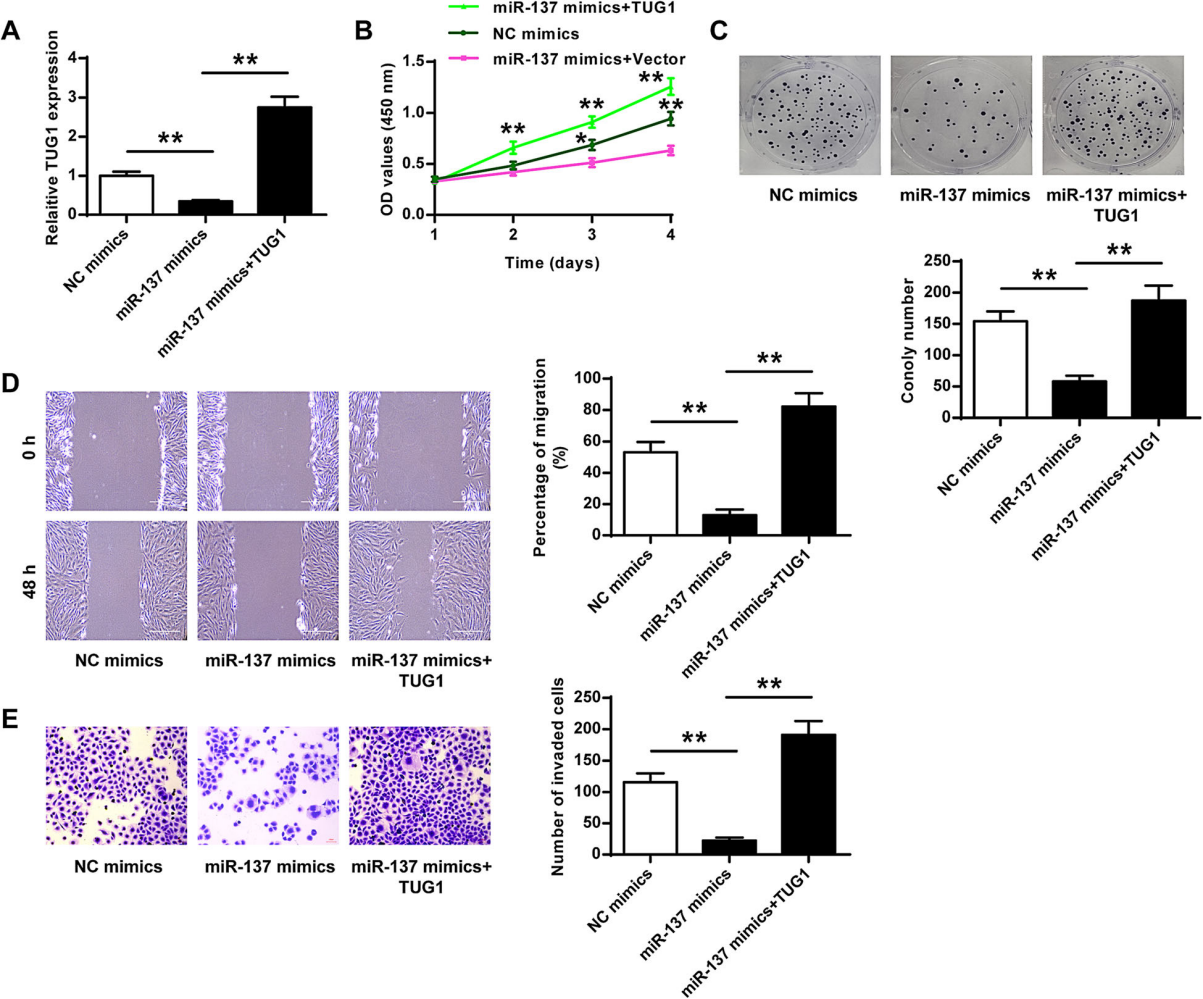
MiR-137 mediated the role of TUG1 in HemECs cells. HemECs were transfected with miR-137 mimics, NC mimics, or co-transfected with miR-137 mimics and TUG1 (the overexpressing vector for TUG1). **A** TUG1 expression was assessed by qRT-PCR. **B, C** Cell proliferation was evaluated by CCK-8 assay (**B**) and colony formation assay (**C**). **D** Cell migration was evaluated by the wound healing assay. **E** Cell invasion was evaluated by Transwell assay. Data were derived from three independent experiments and expressed as mean ± SD. * *P* < 0.05, ** *P* < 0.01

Subsequently, we explored whether miR-137 mediated the role of TUG1 in IH and transfected miR-137 mimics or co-transfected with miR-137 mimics and TUG1 into HemECs. QRT-PCR indicated that transfection of miR-137 mimics significantly reduced TUG1 expression compared with NC mimics (*p* < 0.01), and co-transfection of miR-137 mimics and TUG1 rescued TUG1 expression (*p* < 0.01, Fig. 5A). As shown in Fig. 5B–E, transfection of miR-137 mimics significantly inhibited the proliferation, migration, and invasion of HemECs compared with NC mimics, and co-transfection of miR-137 mimics and TUG1 obviously reversed the inhibitory effect of miR-137 mimics. These results indicated that the role of TUG1 in IH might be partially mediated by miR-137.

### TUG1 knockdown inhibited HemEC proliferation, migration, and invasion by regulating IGFBP5

**Fig. 6:**
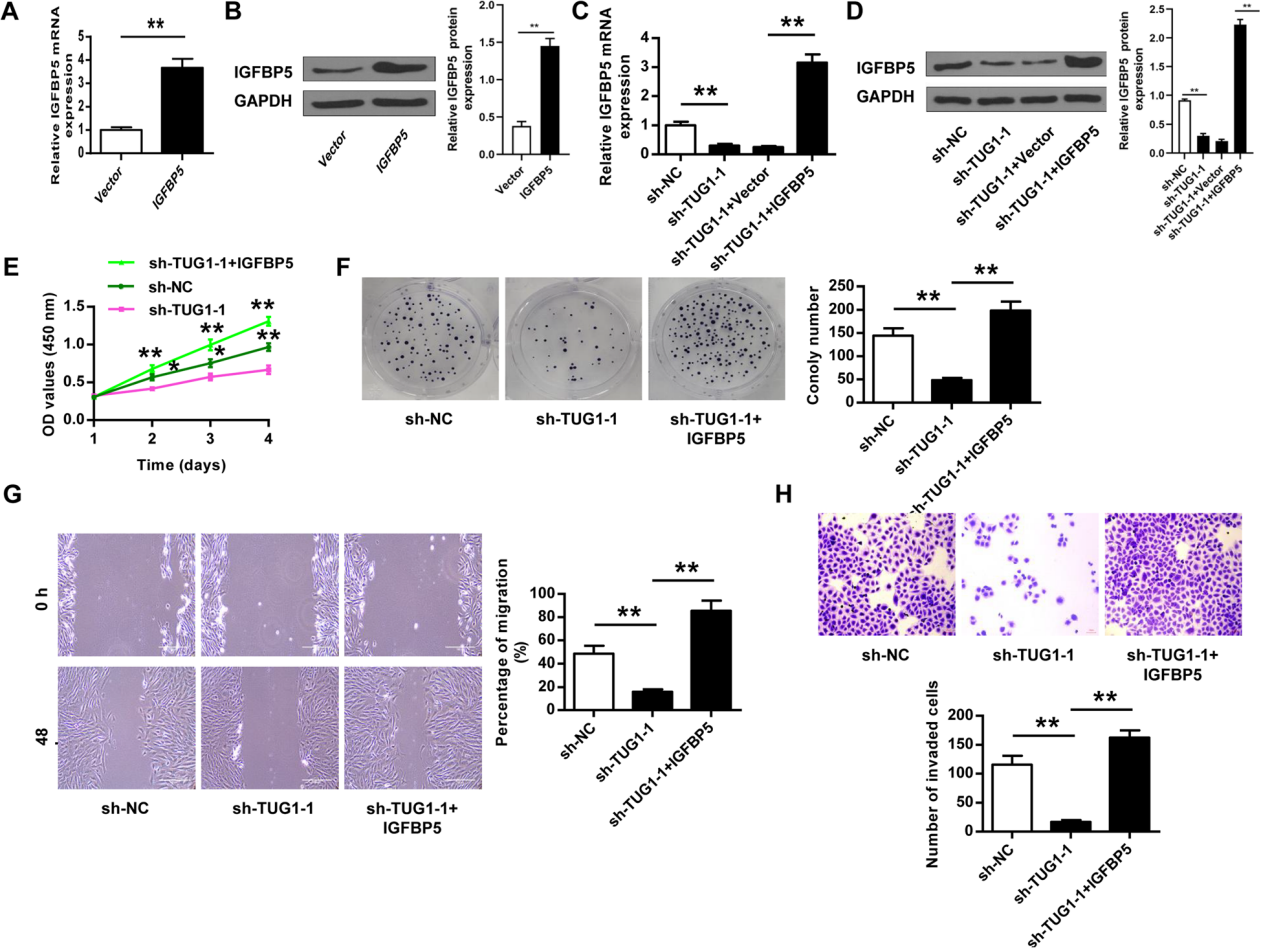
TUG1 knockdown inhibited HemEC proliferation, migration, and invasion via regulating IGFBP5. **A, B** HemECs were transfected with IGFBP5 (the overexpressing vector for IGFBP5) or the empty vector pcDNA3.1. The transfection efficiency was confirmed by qRT-PCR (**A**) and Western blot (**B**). **C–H** HemECs were transfected with sh-TUG1-1, sh-NC, or co-transfected with sh-TUG1-1 and the empty vector pcDNA3.1, or sh-TUG1-1 and IGFBP5 (the overexpressing vector for IGFBP5). IGFBP5 expression was evaluated by qRT-PCR (**C**) and Western blot (**D**). Cell proliferation was evaluated by CCK-8 assay (**E**) and colony formation assay (**F**). **G** Cell migration was evaluated by the wound healing assay. **H** Cell invasion was evaluated by Transwell assay. Data were derived from three independent experiments and expressed as mean ± SD. * *P* < 0.05, ** *P* < 0.01

To investigate whether TUG1 regulated the progression of HemECs by modulating IGFBP5, HemECs were transfected with IGFBP5 and the empty vector pcDNA3.1, and the transfection efficiency was confirmed by qRT-PCR (*p* < 0.01, Fig. 6A) and Western blot (Fig. 6B). In addition, sh-TUG1-1 transfection significantly reduced IGFBP5 expression compared with sh-NC in HemECs (*p* < 0.01), and co-transfection of sh-TUG1-1 and IGFBP5 obviously rescued the induction effects of sh-TUG1-1 on IGFBP5 expression (*p* < 0.01, Fig. 6C and D). Moreover, co-transfection of sh-TUG1-1 and IGFBP5 significantly reversed the inhibitory effects of sh-TUG1-1 on proliferation, migration, and invasion of HemECs (Fig. 6E–H). These results indicated that TUG1 knockdown inhibited the proliferation, migration, and invasion of HemECs by downregulating IGFBP5.

### TUG1 knockdown inhibited the tumorigenesis of HemECs in vivo

**Fig. 7:**
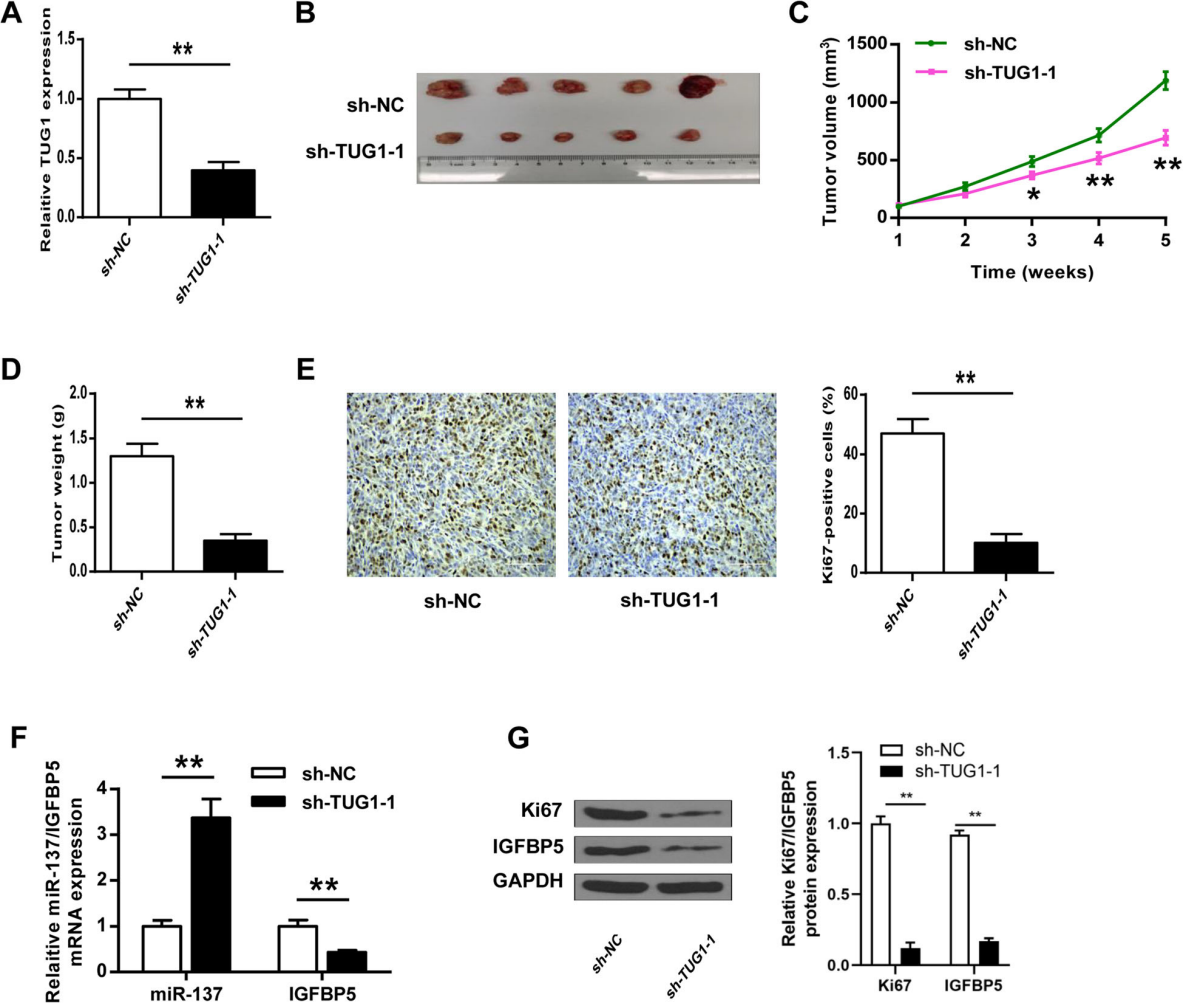
TUG1 knockdown inhibited the tumorigenesis of HemECs in vivo. **A** TUG1 expression in tumor tissues of the mouse model was assessed by qRT-PCR. **B** The representative images of tumors. **C** Tumor volume. **D** Tumor weight. **E** Ki-67 expression in tumor tissues was evaluated by IHC assay using an anti-Ki67 antibody. Magnification, × 200; scale bar = 100 μm. **F** The expression of miR-137 and IGFBP5 in tumor tissues was evaluated by qRT-PCR. **G** The expression of IGFBP5 and Ki-67 in tumor tissues was evaluated by Western blot. Each group included five mice. Data were expressed as mean ± SD. * *P* < 0.05, ** *P* < 0.01

To confirm the oncogenic role of TUG1 in IH progression, an in vivo animal model was established by subcutaneously inoculating sh-TUG1-1-transfected HemECs. TUG1 expression in tumor tissues was evaluated by qRT-PCR, and the results showed that TUG1 level was lower in tumor tissues of the sh-TUG1-1 group than that of sh-NC group (*p* < 0.01, Fig. 7A). As shown in Fig. 7B–D, sh-TUG1-1 obviously reduced tumor volume and tumor weight of mice compared with sh-NC (*p* < 0.03 at day 3, *p* < 0.01 at day 4, *p* < 0.01 at day 5, Fig. 7C; *p* < 0.01, Fig. 7D). Meanwhile, the number of Ki-67 positive cells was significantly reduced in tumor tissues of sh-TUG1-1 group compared with that of sh-NC group (*p* < 0.01, Fig. 7E). In addition, miR-137 expression was significantly increased, and IGFBP5 expression was reduced in tumor tissues of sh-TUG1-1 group compared with that of sh-NC group (*p* < 0.01 for miR137, *p* < 0.01 for IGFBP5, Fig. 7F). IGFBP5 and Ki-67 levels in tumor tissues were confirmed by Western blot (Fig. 7G). These results suggested that TUG1 knockdown inhibited the tumorigenesis of HemECs in vivo by regulating miR-137 and IGFBP5.

## Discussion

As the most common vascular tumors of infancy, IH has become a threat to vision and even life depending on the location and size of the lesion [35] and drawn more attentions to develop efficient therapeutic strategies, which may depend on the well understanding of IH pathogenesis [36]. In the present study, we provided that lncRNA TUG1 might be a novel target for IH treatment and extended the mechanism by which TUG1 affected IH progression by regulating the miR-137/IGFBP5 axis.

Increasing evidences demonstrate the essential roles of lncRNAs in IH progression. For example, MALAT1 is highly expressed in IH tissues, and MALAT1 knockdown inhibits HemEC proliferation, apoptosis, migration, invasion, and tube formation rate [34]. NEAT1 silencing inhibits IH tumorigenesis by competitively sponging miR-33a-5p to activate HIF1alpha/NF-kB signaling pathway [37]. Linc00152 knockdown exerts a potent anti-hemangioma effect by inactivating the Akt/mTOR pathways [38]. However, the effects of more lncRNAs in IH needed to be further explored. Although lncRNA TUG1 has been well studied in many human cancers [39], its role in IH progression has not been explored. In this study, we found that TUG1 was a potential oncogene, and TUG1 knockdown efficiently inhibited HemEC progression both in vitro and in vivo. Interestingly, we found the oncogenic role of TUG1 in IH, suggesting that it might act as a potential therapeutic target for IH.

We identified that TUG1 acted as the sponge of miR-137 and confirmed their relationship using the luciferase reporter assay. Previous studies reported that several miRNAs are direct targets of TUG1 except for miR-137 and mediate the role of TUG1 in human diseases. TUG1 has been identified to sponge miR-145a-5p to affect microglial polarization after oxygen-glucose deprivation [40]. TUG1 alleviates sepsis-induced acute lung injury by targeting miR-34b-5p/GAB1 [41]. In addition, there are some other miRNAs such as miR-148b in ox-LDL-stimulated vascular smooth muscle cells and umbilical vein endothelial cells [42] and miR-145 in papillary thyroid cancer [10]. Function assays revealed that miR-137 overexpression obviously reversed the inhibitory effects of TUG1 knockdown on HemEC proliferation, migration, and invasion. However, the correlation between TUG1 and miR-137 and its role in IH progression have not been reported. Our study revealed that miR-137 was also a target of TUG1 and participated in the function of TUG1 in the tumorigenesis of HemECs. Our study provided a new function of miR-137 in IH progression.

More and more reports have indicated that miRNAs participate in cancer progression mainly via inhibiting the translation of key genes involved in cellular metabolism and phenotypic transition [43]. Their roles in IH have also been widely reported. For example, miR-424 inhibits the proliferation of hemangioma-derived endothelial cells by targeting VEGFR-2 [44]. MiR-424 suppresses cell proliferation, migration, and tube formation capabilities in IH via bFGF/FGFR1 pathway [4]. miR-130a silencing inhibits IH progression by modulating targeting tissue factor pathway inhibitor 2-mediated FAK signaling pathway [45]. We found that IGFBP5 was a target of miR-137 and IGFBP5 overexpression significantly reversed the inhibitory effects of miR-137 mimics and TUG1 knockdown on HemEC proliferation, migration, and invasion. Although IGFBP5 was found to act as a key risk factor in various human diseases in previous studies [26, 46], its role in IH remains unclear. Our study revealed that TUG1 and miR137 participated in IH progression through regulating IGFBP5 expression both in vitro and in vivo and provided that IGFBP5 functioned as a risk gene in IH. Moreover, an in vivo mouse model was used to confirm the potential oncogenic role in IH. However, the signaling pathway(s) involved in the roles of the TUG1/miR-137/IGFBP5 axis in IH need to be further investigated in subsequent experiments.

Our results showed that LncRNA as a sponge by competing for miR-137 binding to regulate the IGFBP5 expression, thereby promoting IH progression. Yang et al. showed that LncRNA MIR31HG is a miR-193b sponge and miR-193b overexpression suppresses MIR31HG expression and function, suggesting that MIR31HG is a target of miR-193b [47]. Interestingly, we also found that miR-137 overexpression suppresses LncRNA TUG1 expression and function, suggesting that LncRNA TUG1 may be a target of miR-137, which needs to be further investigated in subsequent experiments. In addition, we found that TUG1 knockdown inhibited the tumorigenesis of HemECs in vivo, accompanied by miR-137 upregulation and IGFBP5 downregulation in tumor tissues. These results suggested that lncRNA TUG1 depletion suppressed the tumorigenesis of IH by reducing competitively binding of miR-137 to inhibit IGFBP5 expression. Therefore, blocking the TUG1/miR-137/IGFBP5 signaling pathway may represent a potential antitumor therapeutic strategy.

## Conclusions

Our study demonstrated that TUG1 functioned as oncogenic lncRNA in IH by sponging miR-137 to upregulate IGFBP5, providing a novel insight for IH pathogenesis.

## Data Availability

The datasets used and/or analyzed during the current study are available from the corresponding author on reasonable request.

## Abbreviations

IH: Infantile hemangioma
qRT-PCR: Quantitative reverse transcriptase PCR
CCK-8: Cell Counting Kit-8
lncRNAs: Long noncoding RNAs
miRNAs: MicroRNAs
sh-TUG: Short hairpin RNA targeting TUG
IGFBP5: Insulin-like growth factor-binding protein 5
ISH: In situ hybridization
IHC: Immunohistochemistry
TUG1: Taurine upregulated gene 1
